# Periostin: A Matricellular Protein With Multiple Functions in Cancer Development and Progression

**DOI:** 10.3389/fonc.2018.00225

**Published:** 2018-06-12

**Authors:** Laura González-González, Javier Alonso

**Affiliations:** Unidad de Tumores Sólidos Infantiles, Área de Genética Humana, Instituto de Investigación de Enfermedades Raras, Instituto de Salud Carlos III, Madrid, Spain

**Keywords:** periostin, matricellular proteins, cancer, microenvironment, biomarker, cancer hallmarks

## Abstract

Tumor microenvironment is considered nowadays as one of the main players in cancer development and progression. Tumor microenvironment is highly complex and consists of non-tumor cells (i.e., cancer-associated fibroblast, endothelial cells, or infiltrating leukocytes) and a large list of extracellular matrix proteins and soluble factors. The way that microenvironment components interact among them and with the tumor cells is very complex and only partially understood. However, it is now clear that these interactions govern and modulate many of the cancer hallmarks such as cell proliferation, the resistance to death, the differentiation state of tumor cells, their ability to migrate and metastasize, and the immune response against tumor cells. One of the microenvironment components that have emerged in the last years with strength is a heterogeneous group of multifaceted proteins grouped under the name of matricellular proteins. Matricellular proteins are a family of non-structural matrix proteins that regulate a variety of biological processes in normal and pathological situations. Many components of this family such as periostin (POSTN), osteopontin (SPP1), or the CNN family of proteins have been shown to regulate key aspect of tumor biology, including proliferation, invasion, matrix remodeling, and dissemination to pre-metastatic niches in distant organs. Matricellular proteins can be produced by tumor cells themselves or by tumor-associated cells, and their synthesis can be affected by intrinsic and/or extrinsic tumor cell factors. In this review, we will focus on the role of POSTN in the development and progression of cancer. We will describe their functions in normal tissues and the mechanisms involved in their regulation. We will analyze the tumors in which their expression is altered and their usefulness as a biomarker of tumor progression. Finally, we will speculate about future directions for research and therapeutic approaches targeting POSTN.

## Introduction

Hanahan and Weinberg published in the year 2000 an article titled “The hallmarks of cancers” where they summarized by the first time the list of biological capabilities that should be acquired during the development of cancer. The list of such hallmarks included six fundamental biological functions: sustained proliferation signaling, evasion of growth suppression, resistance to cell death, replicative immortality, induction of angiogenesis, and activation of invasion and metastasis (1). Eleven years later, they revisited the concept of cancer hallmarks and included two new enabling characteristics, namely genome instability and mutation and tumor-promoting inflammation, and two emerging hallmarks, namely deregulation of cellular energetics and evasion of immune system (2). In addition, it was also anticipated the important role of tumor microenvironment in the acquisition and modulation of the hallmarks of cancers.

In addition to genetic and epigenetic alterations that occur in cancer cells, and which are in the origin of the hallmarks of cancer, in the last years, there have been accumulated numerous evidences supporting a main role of tumor microenvironment in the modulation, and even induction, of cancer hallmarks (3). As a proof of the interest generated by tumor microenvironment as a key player in cancer development, articles addressing this topic have increased a 400% from year 2000.

Tumor microenvironment can be defined as everything that surrounds tumor cells that are not the tumor cells themselves. Thus, tumor microenvironment is composed of stroma cells (for example, cancer-associated fibroblasts), immune cells that infiltrate the tumor, extracellular matrix (ECM) proteins, and soluble components such as hormones, growth factor, or cytokines. Tumor cells are able to alter the microenvironment and change the properties of the host tissue and vice versa, the composition of the tumor microenvironment influences the behavior of cancer cells (4). In consequence, these cross-talks between cancer cells and the different components of the tumor microenvironment are nowadays considered an essential player in the process that conducts to transformation and cancer.

As mentioned above, one of the main components of the tumor microenvironment is the extracellular cell matrix, which is composed by structural (e.g., collagens) and non-structural proteins that have diverse functions. Within the group of non-structural proteins, there is a subgroup of heterogeneous proteins particularly interesting, named matricellular proteins. The term matricellular proteins was initially proposed by Bornstein et al. (5, 6) to define a group of non-structural ECM proteins that exert several functions by binding to other ECM proteins, growth factor, and cytokines and cells through cell specific receptors in the cell membrane. Matricellular proteins play a central role in the homeostasis of normal tissues regulating cell proliferation and differentiation. These proteins are generally expressed at low levels in most adult tissues, but are highly expressed during inflammation, tissue repair, wound healing, and malignant transformation. Some of the proteins that belong this group of proteins are osteopontin, thrombospondins, members of the CCN family (Cyr61, CCN2, CCN3), tenascins, SPARC, fibulins, or periostin (POSTN) (7).

In this article, we will review the role of the matricellular protein POSTN in cancer development and progression in light of the hallmarks of cancer. First, we will review briefly its molecular characteristics and its functions in normal tissue homeostasis. Next, we described that we know about POSTN expression in solid tumors and their usefulness as a prognostic biomarker. As we will see in detail, POSTN expression is frequently overexpressed by the stromal component of solid tumors and is frequently associated to poor prognosis and metastasis in many cancers. Next, we will review the studies analyzing the effect of POSTN on tumor cells. POSTN interacts with tumor cells through integrin receptors and elicits a plethora of signaling pathways in tumors cells related to cell proliferation, cell survival, or cell migration. Finally, we will speculate about future directions for research and therapeutic approaches targeting POSTN.

## POSTN Structure

Periostin was identified in 1993 as a putative cell adhesion protein produced by a mouse osteoblastic cell line (8). The periostin gene (*POSTN*) in human is located in the long arm of chromosomal 13 (13q13.3) (9) and spans approximately 36 kb. It has 23 exons and encodes for a protein of 836 amino acids with a molecular weight of 93 kDa (8, 10). Alternative splicing of the C-terminal sequence including exons 17–21 gives rise to four POSTN isoforms ranging from 83 to 93 kDa (11). These isoforms have been characterized from different tissues (9, 12–14) and have been shown to be differentially expressed during embryogenesis and bone development (9).

Structurally, POSTN is a multimodular protein composed of a signal peptide, which is necessary for secretion, a small cysteine-rich module (EMI domain) probably involved in the formation of multimers through cysteine disulfide bonds (15), four fasciclin-like domains (FAS1) that interact with integrins (α_v_β3, α_v_β5, α_6_β4) (16), and a hydrophilic C-terminal region known to interact with other ECM proteins such as collagens, fibronectin, tenascin C, or heparin (15, 17). Interesting, the FAS1 domains of human POSTN contain vitamin K-dependent γ-carboxyglutamic acid (Gla) residues (18), which are found in a small group of proteins called Gla-containing proteins. This group of particular proteins includes many coagulation and anticoagulation factors as well as bone-associated proteins such as osteocalcin (bone gamma-carboxyglutamate protein) and matrix-gla protein (MGP) (19). These proteins suffer posttranslational modifications by γ-glutamyl carboxylase (a vitamin K-dependent enzyme) on specific glutamyl amino acid residues (Glu) to produce γ-carboxyglutamic amino acid residues (Gla) (20, 21). The main known function of γ-carboxyglutamic acid residues is their ability to bind divalent cations such as calcium. Hence, Gla-containing proteins play important roles in the regulation of coagulation cascade and bone homeostasis (22, 23).

## Expression and Function of POSTN in Normal Tissues

Periostin is preferentially expressed in the periosteum, hence its name. The periosteum is a specialized membrane, which covers the outer surface of bones and is responsible for growth in diameter of bone and for the cortical thickness. The activity of the periosteum is particularly elevated during the phases of embryonic development and body growth, although during the adult life, it also contributes to determinate bone diameter and, subsequently, bone strength (24, 25). Interestingly, POSTN is also expressed in other connective tissues rich in collagens undergone to mechanical stress such as the periodontal ligament (a specialized structure of the teeth) (26), heart valves (27), and tendons (28). During cardiac development, POSTN is highly expressed by embryonic fibroblasts (29) and pericardial cells that cover the embryonic heart (30), but not by cells of the cardiomyocyte lineages (31). In the bone, POSTN mRNA has been detected by *in situ* hybridization in pre-osteoblast cells (9).

As described above, POSTN is able to interact with cells through its FAS1 domains and ECM proteins through its N-terminal EMI domain and C-terminal region. These properties make POSTN a key player in the regulation of cell behavior and organization of the ECM. POSTN has been shown to bind integrins αvβ3 and αvβ5 in osteoblasts and several types of normal and cancer cells where it elicits activation of FAK, PI3-Kinase, and AKT signaling pathways (32–34). These findings suggest that POSTN can act as a prosurvival protein in many cellular contexts.

Periostin plays an important role in ECM structure and organization and particularly in collagen assembly. Collagen cross-linking is a natural process essential to provide stability to collagen-rich connective tissues. Two key elements in this process are BMP-1 and lysyl oxidase (LOX) (35). Briefly, BMP-1 cleavages the inactive form of LOX to produce the active LOX enzyme, which in turn catalyzes the covalent cross-linking of collagen molecules (35). Interestingly, POSTN binds BMP-1 and collagen I through its FAS1 domains and N-terminal EMI domain, respectively, and thus act as a key player in this process, serving as a scaffold for BMP-1 and collagens to accelerate collagen cross-linking (35). The importance of POSTN in collagen cross-linking is also supported by POSTN knockout animal models. Thus, POSTN null mice exhibit aberrant collagen fibrillogenesis in the periosteum and a decrease in collagen cross-linking observed in skin, tendons, and heart (36).

The function of the Gla residues is, however, much less known. Remarkably, POSTN has 28 glutamyl amino acid residues (Glu) that could be posttranslationally modified to generate γ-carboxyglutamic amino acid residues (Gla) (18). The high number of potential Gla residues present in POSTN contrasts with the number of Gla residues contained in others Gla proteins of the bone such as osteocalcin and matrix Gla protein, which have 3 and 5 Gla residues, respectively. Coutu et al. studied the form of POSTN (carboxylated vs uncarboxylated) that was secreted by adipocytes, chondrocytes, and osteoblasts differentiated from mesenchymal stem cells. They found that undifferentiated human mesenchymal cells and also differentiated adipocytes and osteoblasts synthetized carboxylated POSTN while no POSTN was detected in cells undergone chondrogenic differentiation. Interestingly, carboxylated POSTN was detected in the conditioned medium of undifferentiated human mesenchymal cells and differentiated adipocytes but not in the conditioned medium derived from differentiated osteoblasts. In the latter case, POSTN was found to be abundantly deposited in bone nodules produced *in vitro*, indicating that osteoblasts are able to produce carboxylated POSTN that is efficiently sequestered within the ECM in a calcium-dependent manner (18).

## Regulation of POSTN Expression

Periostin expression is transcriptionally regulated by several transcription factors that are themselves involved in the commitment of pluripotent mesenchymal cells to cells of the osteoblastic lineage. Several studies indicate that the transcription factors Twist-1 and -2 are involved directly in the regulation of POSTN expression. For example, Oshima et al. detected a “Twist box” response element in the POSTN promoter and demonstrated that Twist can bind this Twist-box sequence and activate the POSTN promoter in a reporter assay (37). A direct demonstration about the involving of the Twist family of transcription factors in the regulation of POSTN expression comes from observations done in a rare autosomal recessive disease named Setleis syndrome (OMIM 227260) characterized by abnormal facial development. Franco et al. demonstrated that patients with this disease harbor mutations in the bHLH transcription factor *TWIST2* and that expression of POSTN was severely downregulated in fibroblasts derived from patients with this disease. Reporter gene assays and ChIP assays demonstrate that wild-type *TWIST2* was able to induce POSTN promoter, while the mutant *TWIST2* found in the patients was not, providing a direct link between POSTN expression and *TWIST2* (38). Other studies also suggest a relationship between Twist and POSTN. Thus, Hu et al. re-analyzed public data obtained from The Cancer Genome Atlas dataset and observed that POSTN expression levels correlated with Twist and Snail expression in lung cancer specimens (39). In addition, a Twist shRNA was also shown to be able to inhibit POSTN expression in prostate cancer cell lines (40).

c-Fos/c-Jun (AP-1) are other transcriptional factors that can be involved in the regulation of POSTN expression. Kashima et al. analyzed the expression of POSTN in bones from patients with fibrous dysplasia, a benign bone disease characterized by high expression of transcriptional factors such as c-Fos/c-Jun. Immunohistochemistry and *in situ* hybridization studies revealed that POSTN was expressed in the fibrous component of fibrous dysplasia lesions correlating with c-Fos expression. These authors also analyzed POSTN levels in the sclerotic lesions developed in transgenic mice overexpressing c-fos, which are similar to those observed in fibrous dysplasia. In these lesions, all transformed osteoblasts expressed high levels of POSTN whereas normal osteoblasts did not, providing a relationship between c-fos overexpression and POSTN expression (41). Other transcription factor that has been shown to regulate POSTN expression is p73. Landre et al. showed, using ChIP assays, that the transcription factor p73 binds POSTN promoter. They demonstrated that p73 conferred an invasive phenotype to glioblastoma cells, which was mediated by activation of POSTN. In fact, POSTN overexpression was sufficient to rescue the invasive phenotype of glioblastoma cells after p73 knockdown (42). Overexpression of the transcription factors Slug and Sox9 induced tenascin-C and POSTN expression.

There are several hormones and growth factors that have been shown to regulate POSTN expression, which are also involved in bone homeostasis. For example, several studies indicate that parathyroid hormone affects POSTN expression (43–46) and *vice versa* (45). Estrogens are other type of hormones that have been shown to regulate POSTN expression. So, estradiol induces POSTN mRNA expression in primary human periodontal ligament cells through stimulation of estrogen receptor B (47). Finally, some cytokines and growth factors also regulate the levels of POSTN. For instance, TGF-β induces POSTN expression in osteoblasts (26), human periodontal ligament cells (48–50), gingival fibroblasts (51, 52), and mesangial cells of the kidney (53). BMP-2 induces POSTN expression in a murine mesenchymal progenitor cell line (54) and in atrioventricular mesenchymal cells (55). Angiotensin II induces POSTN expression in rat cardiac fibroblasts (56) and vascular smooth muscle cells (57). Finally, several studies indicate that other growth factors and cytokines such as PDGF, bFGF, TNFα, IL-4, IL-13, or Oncostatin M have been shown to induce POSTN expression in determined cells and/or animal models (57–61).

## Tumor POSTN as a Biomarker

Periostin expression is deregulated in several pathologies such as inflammation, tissue repair, and malignant transformation (6). Next, we will review the current knowledge about deregulation of POSTN expression in cancer. In general, high POSTN levels are usually associated with a more aggressive tumor behavior, advanced stage or poor prognosis, suggesting that POSTN levels could be a useful prognostic biomarker (Table 1).

**Table 1:** Correlation between periostin expression levels and clinical parameters in solid tumors.

Cancer	High expression in	Is associated with (1)	Reference
Prostate cancer	Stroma	OS (poor prognosis)	(62)
	Stroma	PFS (poor prognosis)	(63)
	Stroma	High gleason score	(63–65)
	Stroma	Advanced stage	(63)

Lung cancer	Stroma	OS (poor prognosis)	(66)
	Stroma	OS (poor prognosis)	(67)

Pancreatic cancer	Stroma and cancer epithelial cells	OS and PFS (poor prognosis)	(68)
	Stroma produced by pancreatic stellate cells	Histological grade (poor prognosis)	(69)

Ovarian cancer	Stroma and cancer epithelial cells	OS and PFS (poor prognosis)	(70)
	Stroma and cancer epithelial cells	Advanced stages and cancer recurrence	(71)

Breast cancer	Stroma and cancer epithelial cells	OS and PFS (poor prognosis)	(72, 73)
	Cancer-associated fibroblasts	OS (poor prognosis)	(74)

Colorectal cancer	Stroma	OS and PFS (poor prognosis)	(75)
	Cancer epithelial cells	Advanced stage and metastasis	(32, 76)

Hepatocellular carcinoma	Cancer epithelial cells	OS and PFS (poor prognosis)	(77)
	Stroma and cancer epithelial cells	Tumor grade	(77)
	Stroma	Increased microvascular invasion (poor prognosis)	(78)

Bladder cancer	Stroma	Not studied	(14)
	Extracellular vesicles	Tumor stage (poor prognosis)	(79)

Osteosarcoma	Tumor	OS and PFS (poor prognosis)	(80)

*(1) OS, overall survival; PFS, progression-free survival*.

### Prostate Cancer

Several studies have demonstrated that POSTN expression is altered in prostate cancer. Nuzzo et al. focused their attention on the distribution of POSTN in tumor tissues. They showed that POSTN could be detected in the tumor stroma, cancer epithelial cells, as well as in peritumoral areas. High expression of POSTN in the stromal component was associated with shorter survival while lower POSTN expression in cancer epithelial cells was significantly correlated with shorter progression-free survival, suggesting that patients with high levels of POSTN in the stroma component and low levels in cancer epithelial cells had worse prognosis (62). Tischler et al. also showed that the strong POSTN expression observed in tumor stroma was associated with a shorter progression-free survival (63). Other studies have shown that high expression of POSTN in tumor stroma is related to Gleason score (63, 65), the stage of the tumor (63), and the degree of malignancy (64). High POSTN expression was associated with high fibronectin expression and low expression of integrin α4 in metastatic castration-resistant prostate cancer patients (81).

### Lung Cancer

In non-small cell lung cancer (NSCLC), POSTN has been found in mesenchymal areas in tumor stroma, but not in the cancer epithelial cells themselves (66). Studies carried out in patients have revealed that high expression of POSTN in tumor stroma was significantly associated with shorter overall survival rates (66). A study accomplished by Nitsche et al. showed that POSTN expression was an independent prognostic factor in a multivariate analysis that included POSTN expression, histological tumor subtype, tumor stage, lymph node involvement, and resection status (67).

### Pancreatic Cancer

In pancreatic ductal adenocarcinoma (PDAC), POSTN has been detected in cancer epithelial cells, pancreatic stellate cells, and tumor stroma. Ben et al. showed that high POSTN expression in stroma and cancer epithelial cells compared with adjacent tissue were indicative of poor prognosis (68). In other study, it was shown that high POSTN expression in tumor stroma correlated with pathological grade and with survival rate in PDAC patients (69).

### Ovarian Cancer

Periostin is expressed a high levels in tumor stroma and in cancer epithelial cells in ovarian cancer. In a study, high POSTN expression in stroma was associated significantly with lower overall survival and progression-free survival (70). By contrast, high POSTN levels in cancer epithelial cells did not show significant prognostic value compared with patients with lower POSTN expression in the cancer cells. However, in other study, patients with high POSTN expression in both, stroma and cancer epithelial cells had the shortest overall survival and progression-free survival (70). High levels of POSTN have been also correlated with advanced clinical late stages (III/IV) and cancer recurrence (71).

### Breast Cancer

Breast cancer is characterized by high POSTN expression in cancer epithelial cells when compared with normal tissue. The elevated expression of POSTN has been associated with poor progression free and overall survival (73). Lambert et al. studied the relationship between the expression of POSTN in cancer stem cells and the prognosis of the patients. Their studies revealed that high POSTN expression in these cells was associated with reduced relapse-free survival in basal-like type but not in breast cancers of the luminal type (72). In addition to cancer cells, POSTN is also present in cancer-associated fibroblasts (CAFs). In this case, the higher levels of POSTN detected in CAF are associated with the malignancy grade of tumors, suggesting that POSTN secreted by CAFs could be a key element in breast cancer progression (74).

### Colorectal Carcinoma

In colorectal carcinoma, POSTN expression is increased compared with normal tissues. According to immunohistochemical analysis, POSTN is expressed by CAFs and secreted to the stroma (75). Several independent studies have shown a correlation between POSTN expression and disease aggressiveness. For example, Bao et al. showed that expression levels of POSTN mRNA were higher in liver metastasis than in the primary tumor derived from the same patients (32). A multivariate analysis described in Wu et al. showed that the presence of liver metastasis correlated with histological type, lymph node metastasis, TNM stage, and POSTN levels (76). Moreover, Li et al. described a positive correlation between high POSTN expression and tumor size, grade of cell differentiation, lymph node metastasis, serosal invasion, clinical stage, and 5-year survival rates (82). More recently, Xu et al. described a study carried out in two independent cohorts of patients showing that medium- and high-stromal POSTN expression predicted poor prognosis in patients with colorectal carcinoma (83). Finally, a multivariate analysis performed in patients with colorectal carcinoma showed that high expression of stromal POSTN was an independent prognostic biomarker of poor overall survival and progression-free survival (75). Taken together, these findings strongly support an important role for POSTN in the development, progression, and metastatic dissemination of colorectal cancer.

### Liver Cancer

A strong POSTN expression has been observed in cancer epithelial cells and tumor stroma in hepatocellular carcinoma. Riener et al. showed that POSTN expression in cancer epithelial cells was associated with reduced overall survival and correlated with tumor grade (77). In other study, POSTN expression was associated with tumor nodules, microvascular invasion, Edmodson grade, TNM stage, and higher levels of vascular endothelial growth factor (VEGF) expression. Besides, the expression of POSTN was found to be an independent prognostic factor for predicting overall and disease-free survival in a multivariate analysis (84). More recently, Jang et al. showed that high POSTN expression in hepatocellular carcinoma was correlated with microvascular invasion, and advanced stage disease and patients with high perisotin expression had significantly lower overall survival rates suggesting a relationship between POSTN expression and poor prognosis (78). Utispan et al. showed that POSTN is over-expressed by CAFs in intrahepatic cholangiocarcinoma but not by cancer cells or immune infiltrating cells. Patients with higher levels of POSTN had shorter survival rates than those with lower levels (85).

### Bladder Cancer

Unlike most tumors, POSTN expression appears to be downregulated in bladder cancer compared with normal tissue. Immunohistochemically, analysis demonstrated a strong staining of POSTN in the stroma of normal bladder, while it was mostly attenuated in bladder cancer tissues. Strikingly, POSTN downregulation correlated inversely with tumor grade. Thus, while POSTN expression was detected in 100% normal bladder tissues, only a 33% of grade 3 bladder cancer expressed POSTN (86). Normal bladder tissues expressed the canonical isoform of POSTN as well as others alternatively spliced mRNAs. By contrast, in bladder cancer tissues, the canonical POSTN isoform is not expressed and only some alternatively spliced isoforms can be detected. Interestingly, the alternative isoforms expressed in cancer tissues did not show tumor suppressor activity as the canonical isoform did (14, 86). These findings suggest that loss of canonical POSTN can be related with the development of bladder cancer.

However, other studies suggest an opposite role for POSTN in the development of bladder cancer. In muscle-invasive bladder cancer, an aggressive malignancy with high mortality, high POSTN expression correlated with worse prognosis. These tumors produce extracellular vesicles, which have been shown to promote cancer progression. Extracellular vesicles isolated from urine of patients had markedly higher levels of POSTN than controls, indicating that POSTN could be a potential urinary biomarker of cancer progression (79).

### Osteosarcoma

Hu et al. analyzed the expression of POSTN and VEGF in patients with osteosarcoma. The histopathological and immunohistochemically analysis showed that POSTN expression was higher in osteosarcoma than in osteochondroma and correlated with VEGF expression, histological subtype, and tumor size (80). Furthermore, patients who showed higher levels of POSTN had a poorer prognosis than those with lower POSTN expression. Overall survival was twofold higher in patients with lower POSTN compared with patients with high POSTN expression (80).

### Other Cancers

In esophageal squamous cell carcinoma, high POSTN expression correlated with poor prognosis and shorter overall survival. In a multivariate analysis including tumor differentiation, venous invasion, TNM stage, and other parameters, POSTN expression level was identified as an independent prognostic factor (87).

Morra et al. analyzed the levels of POSTN in renal cell carcinoma and showed that POSTN was expressed in mesenchymal cells, cancer epithelial cells, and tumor stroma. Higher levels of POSTN in cancer epithelial cells correlated with the presence of sarcomatoid differentiation, higher tumor stage, lymph node metastases, and poor overall survival. The authors suggest that POSTN expression in cancer epithelial cells may contribute to sarcomatoid differentiation and a more aggressive behavior of renal cell carcinoma (88).

## Serum POSTN Levels as a Biomarker of Poor Prognosis

Since POSTN levels in tumor tissues show relationship with prognosis in many cancers, several studies have analyzed if POSTN levels in serum correlate with cancer prognosis. In fact, increased POSTN levels have been detected in serum of patients with some malignant tumors suggesting that it could be an effective biomarker, both for diagnosis and survival prediction (summarized in Table 2).

**Table 2:** Serum periostin levels as a prognostic factor.

Cancer	Diagnostic/prognostic value	Reference
Lung cancer	Associated to bone metastasis	(89)
	Associated to bone metastasis	(90)
	Response to chemotherapy	
	Associated to poor survival	
	Associated to poor survival (independent prognostic factor)	(91)

Breast cancer	Associated to poor survival (in some subgroups)	(92)
	Associated to bone metastasis	(93)

Colorectal cancer	Associated to poor prognosis	(94)
	Associated to metastasis	

Hepatocellular carcinoma	Associated to poor prognosis	(95)

Cholangiocarcinoma	Associated to poor prognosis (independent prognostic factor)	(96)
	Diagnostic value	(97)

### Lung Cancer

Xu et al. analyzed the levels of serum POSTN in a large cohort of 296 patients with NSCLC. The levels of serum POSTN in patients with cancer were significantly elevated compared to healthy controls and patients with benign lung disease. Patients with the higher serum POSTN levels had a poor progression-free survival and overall survival than patients with lower levels. POSTN levels were shown to be an independent prognostic factor (91). In other similar study, Zhang et al. showed that serum POSTN levels were also significantly higher in NSCLC compared to patients with benign lung disease and healthy controls (90). It was also shown that serum POSTN levels were significantly associated with the presence of bone metastasis. Interestingly, POSTN levels before and after chemotherapy correlated significantly with prognosis suggesting that POSTN levels could be able to predict chemotherapy response and survival in patients with advanced NSCLC (90). Che et al. studied the levels of serum POSTN in patients with lung cancer with or without bone metastasis. Interestingly, patients with bone metastasis presented higher levels of POSTN compared to patients without bone metastasis and healthy controls (89). Other study, however, found no relationship between serum POSTN levels with clinic and pathologic parameters, although serum POSTN in the lung cancer patients were elevated compared with healthy controls (98).

### Breast Cancer

The relationship between serum POSTN levels and prognosis in breast cancer has also been studied. Sasaki et al. found increased levels of serum POSTN in breast cancer patients with bone metastasis compared to patients without metastasis, although no correlation with other prognostic factors such as clinical stage and lymph node metastasis was found (93). More recently, Nuzzo et al. analyzed the serum POSTN levels in patients with early breast cancer who had received curative surgery and found some association between POSTN and prognosis only in some subgroups of patients. Specifically, they found a correlation between higher POSTN serum levels and mortality in patients with node-negative disease and in patients who did not receive adjuvant therapy (92).

### Colorectal Cancer

Ben et al. showed that serum POSTN levels were significantly elevated in colorectal cancer patients compared to healthy controls or patients with benign colorectal polyps or adenomas. POSTN levels in serum correlated with mRNA expression levels in the tumor and also with tumor size, since serum POSTN levels undergone a significant decrease after surgery. Serum POSTN levels correlated significantly with distant metastasis, advanced-stage disease, and poor prognosis, suggesting that circulating POSTN may be of clinical value in identifying patients who could be at risk of metastasis and progression (94). By contrast, a recent study comparing serum POSTN levels between patients with and without bone metastasis not found significant differences between both groups although, in this study, the number of patients with colorectal cancer was low (99).

### Liver Cancer

Serum POSTN levels have been shown to be increased in patients with hepatocellular carcinoma compared to healthy controls or patients with no cancerous liver diseases. Elevated serum POSTN were associated with poor overall survival and release-free survival. In multivariate analysis, POSTN was considered an independent prognostic marker for predicting survival (95). Fujimoto et al. analyzed the levels of serum POSTN in intrahepatic cholangiocarcinoma patients and observed that these were significantly higher in these patients compared to normal controls and patients with liver cirrhosis, hepatocellular carcinoma, and other malignancies, suggesting that serum POSTN levels could be useful to distinguish between cholangiocarcinoma and other hepatic malignancies (97). In a recent study, similar results were observed. Thus, serum POSTN levels in patients with cholangiocarcinoma were significantly increased compared with that in healthy controls, patients with benign liver diseases, and even patients with breast cancer, indicating that serum POSTN levels could be used as a diagnostic biomarker (96). Furthermore, high levels of serum POSTN, as well as tissue POSTN levels, were significantly associated with reduced survival rate. Serum POSTN was identified as an independent prognostic factor suggesting that it could be used as a marker of poor prognosis in patients with cholangiocarcinoma (96).

## POSTN and Resistance to Treatment

Interestingly, several studies have showed that POSTN could play an important role in the resistance to treatment in some types of solid tumors (summarized in Table 3).

**Table 3:** Periostin and resistance to chemotherapy.

Cancer (cell lines)	Drug	Evidences	Pathways involved	Reference
Pancreatic cancer (SW1990, Panc-1)	Gemcitabine	*In vitro, in vivo* (xenograft)	Erk, Akt	(100)

Non-small lung cancer (A549)	Cisplatin	*In vitro, in vivo* (xenograft)	Akt, Stat3, Survivin	(101)

Ovarian cancer (A2780)	Cisplatin	*In vitro*, patients	Akt	(70)

Ovarian cancer (ES-2)	Carboplatin Paclitaxel	*In vitro*, patients	Not studied	(102)

Glioma (GSC272, GSC11)	Bevacizumab	*In vitro, in vivo* (xenograft)	TGFβ, hypoxia-inducible factor-1α, Stat3	(103)

Colon cancer (SW480, HT-29)	Oxaliplatin	*In vitro*	Akt, survivin	(104)
	5-fluorouracil			

Gastric cancer (SGC-7901)	Cisplatin	*In vitro*	Bax, p53, Bcl-2, Akt	(105)
	5-fluorouracil			

For instance, Liu et al. showed that POSTN exerts a significant role in the resistance of pancreatic cancer cells to gemcitabine. In this study, it was shown that silencing of POSTN gene in pancreatic cancer cells sensitize gemcitabine-resistant cells to gemcitabine treatment both *in vitro* and *in vivo*, suggesting that POSTN targeting could be a new therapeutic approach to overcome gemcitabine resistance in pancreatic cancer (100).

Hu et al. studied the effect of POSTN on cisplatin resistance in the A549 NSCLC cells. It was shown that POSTN overexpression makes these cells more resistant to cisplatin-induced apoptosis, while POSTN knockdown sensitive these cells to cisplatin. These results indicate that POSTN induces cisplatin-resistance in non-small lung cancer and thus it could represent a new target for overcoming cisplatin-resistance (101).

Sung et al. also found a relationship between cisplatin resistance and POSTN in patients with ovarian carcinoma. In this case, patients with high levels of stromal POSTN showed cisplatin resistance compared to those with lower levels. *In vitro*, POSTN treatment of A2780 ovarian adenocarcinoma cells induced cisplatin-resistance and activation of the Akt pathway, which was reverted by incubation of the cells with an Akt inhibitor (70). Similar results were also observed by Ryner et al. in a large cohort of ovarian cancer patients (102). In this work, gene expression profile identified a specific gene signature associated to chemoresistant tumors, where POSTN was one of the three highest ranked genes. Furthermore, recombinant POSTN was able to promote resistance to carboplatin and paclitaxel in chemosensitive ovarian cells *in vitro* (102).

Park et al. analyzed the role of POSTN in the resistance to antiangiogenic therapy in glioma stem cells. Using mouse xenograft models of human glioma, they demonstrated that animals treated with bevacizumab expressed higher levels of POSTN than tumors derived from untreated animals. Interestingly, POSTN knockdown increased the median survival of the animals and the effect of POSTN knockdown in combination with bevacizumab was synergic (103). POSTN expression was associated with increased expression of TGFβ1 and hypoxia-inducible factor-1α in glioma stem cells, which explain the effect of POSTN on the resistance to antiangiogenic therapy in these cells.

Treatment of SW480 and HT-29 colon cancer cells with oxaliplatin or 5-fluorouracil increased the levels of POSTN mRNA and protein. POSTN knockdown increased significantly the sensibility of these cells to drug-induced apoptosis, which correlated with a reduction of survivin levels, linking POSTN to the antiapoptotic protein survivin. Conversely, POSTN overexpression increased survivin levels and Akt activation. All together, these results suggest that POSTN induces chemoresistance in colon cancer cells through activation of the PI3K/Akt/survivin pathway (104).

Similar results were also observed in gastric cancer cell lines. In this case, overexpression of POSTN in SGC-7901 cells rendered these cells more resistant to cisplatin or 5-fluorouracil-induced apoptosis. Cells overexpressing POSTN showed a reduction in the levels of Bax and p53 proteins and an increase of Bcl-2 proteins and Akt phosphorylation. Restoration of p53 expression or treatment of the cells with an inhibitor of Akt restored drug sensibility (105).

The molecular mechanism underlying the POSTN-mediated resistance to chemotherapy is largely unknown. Although activation of survival pathways seems to play an important role in the resistance to cancer drugs, other mechanisms, such as those mediated for the family of ATP-binding cassette proteins (ABC family) involved in the acquisition of the multiple drug resistance phenotype in cancer must be taken into consideration. In this sense, POSTN expression has been shown to correlate with some members of the ABC family in some cancer cells (106). Clearly, more studies are necessary to confirm if there is a relationship between POSTN expression and ABC proteins and to determine its contribution to chemotherapy resistance.

## POSTN and the Hallmarks of Cancer

**Figure 1:**
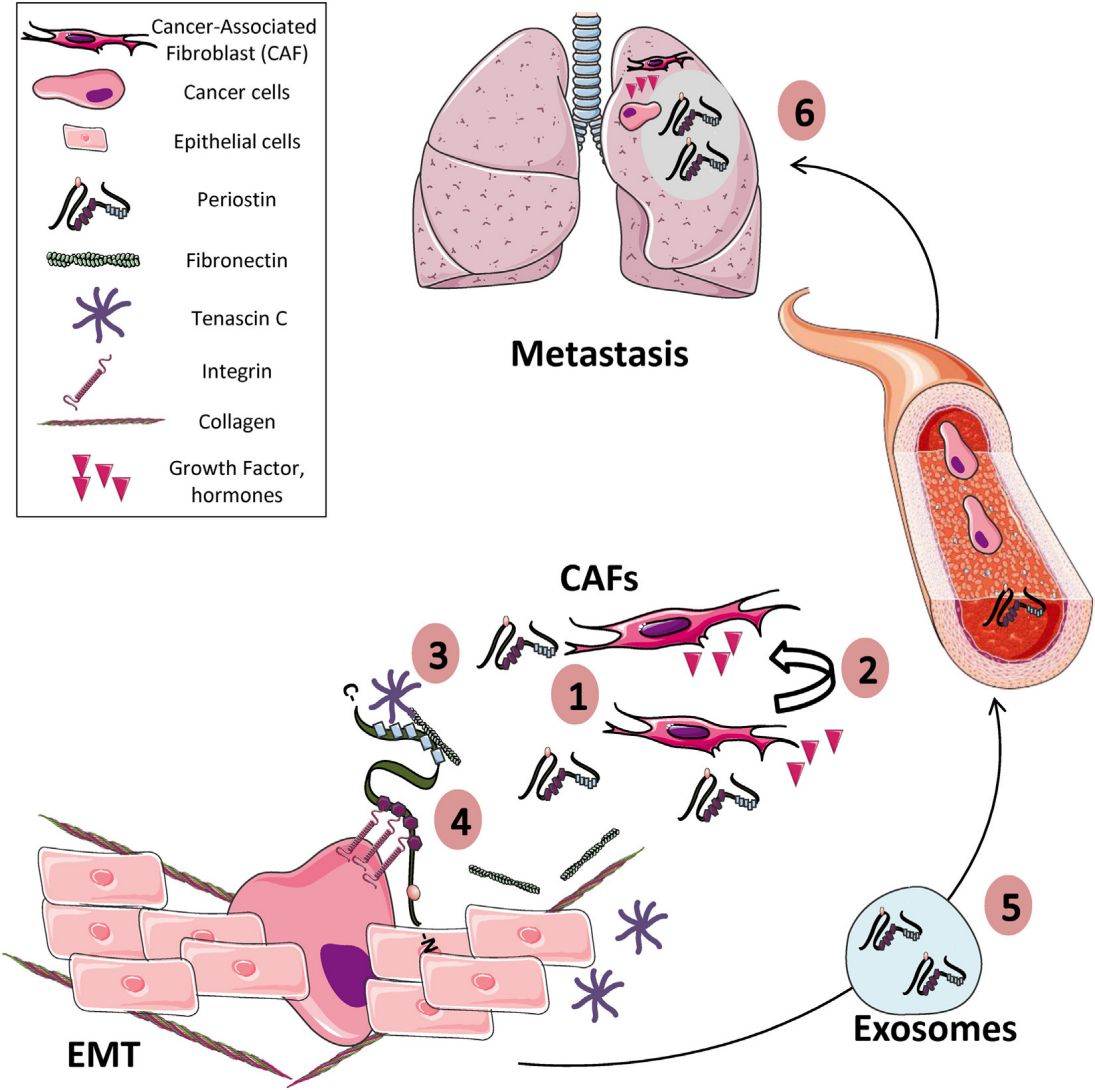
Periostin (POSTN) and the hallmarks of cancer. (1) POSTN is overexpressed in many solid tumors, mainly by stromal cells such as cancer-associated fibroblasts and to a lesser extent by tumor cells themselves. (2) Both paracrine and autocrine signals can stimulate the expression of POSTN by stromal and tumor cells. (3) Extracellularly secreted POSTN interacts with others extracellular matrix (ECM) proteins (e.g., collagens, tenascin C, fibronectin) contributing to produce a tumor-receptive ECM, by modulating, for example, collagen cross-linking. (4) In addition, POSTN interacts with integrin receptors present in the membrane of cancer cells, promoting cell proliferation, cell survival, epithelial–mesenchymal transition (EMT), and migration. (5) POSTN can be transported into exosomes produced from stromal and tumor cells to distant sites in other tissues (e.g., lung, liver, or bone) or produced by CAFs (6) where it contributes to prepare the metastatic niche before the arrival of tumor cells.

The deregulation of POSTN expression in many cancers suggests that it plays an important role in cancer development and progression. In addition, there are strong experimental evidences that involve POSTN in the acquisition of many hallmarks of cancer, such as proliferation, invasion and metastasis, angiogenesis, or cell survival (Figure 1; Table 4).

**Table 4:** Effect of periostin (POSTN) on tumor cells.

Cancer	Cell line	Effect of POSTN	Reference
Clear renal cancer	A498, 786-0	Induces proliferation	(107)
	786-O, ACHN	Induces migration and invasion	(108)

Lung cancer	A549	Induces proliferation and migration	(98)
	CL1, CL1-5	Induces epithelial–mesenchymal transition (EMT)	(80)

Gastric cancer	OCUM-2MLN	Induces proliferation	(109)
	OCUM-12		

Melanoma	NHDF	Induces proliferation	(110)
	SP2/O, SKMEL-28	Induces angiogenesis	(111)
	B6-BL6, B16	Induces metastasis	(112)
	MC3T3-C1		

Prostate cancer	RWPE-1, TA2	Induces EMT	(64)
	PC3, DU145	Induces proliferation, invasion, and migration	(40)

Bladder cancer	SBT31A, T24	Suppresses EMT and invasion	(113)

Ovarian cancer	HOSE, CSOC, SK-OV-3	Induces adhesion and migration	(13)
	A2780	Induces proliferation	(71).

Colorectal cancer	CX-1NS	Induces metastasis (preventing stress-induced apoptosis)	(32)

Esophageal cancer	EPC2	Induces invasion	(114–116)

Glioblastoma	U87, T98G, and A172	Induces proliferation, migration, and invasion	(117)
	GSC and NSTCs	Promotes malignant growth	(118)

Breast cancer	MCF7	Induces angiogenesis	(119)
	MCF7, MDA-MB-231, 67NR, 4T1	Induces metastasis	(120)

Head and neck cancer	HN4, HN6, HN13, HN30	Induces metastasis	(121)
	HNSCC	Induces lymphoangiogenesis	(122)

Oral squamous cell carcinoma	Rca-B, Rca-T	Induces metastasis	(121)

Hepatocellular carcinoma	SMMC7721, Hep3B	Resistance to hipoxia	(123)

Pancreatic cancer	AsPC-1, SW-1990, BxPC-3, Panc-1	Induces proliferation and invasion	(68)

### Sustaining Proliferative Signals

Periostin has been shown to induce proliferation in several tumor cell lines. For example, overexpression of POSTN in the A549 lung cancer cell line promoted cell proliferation and migration by inducing vimentin and N-cadherin expression and downregulating E-cadherin expression (98). Kikuchi et al. showed that POSTN increased proliferation of gastric cancer cell lines, concomitantly with the activation of ERK pathway. Interestingly, orthotopic xenograft tumors carried out with one gastric cancer cell line grew more slowly in Postn (^−/−^) mice than in wild-type mice, suggesting that POSTN produced by CAFs favor the establishment of a growth-supportive microenvironment for gastric cancer (109). In addition, Kotobuki et al. showed that recombinant POSTN and POSTN derived from normal human dermal fibroblasts induced melanoma cell proliferation by activating the MAPK signaling pathway (109, 110).

### Activating Invasion and Metastasis

Periostin is involved in invasion and metastasis by regulating key processes such as epithelial–mesenchymal transition (EMT). Briefly, EMT is a physiological process, first discovered in embryonic development, and later observed also during tumor progression, consisting in the transition of epithelial cells to cells with mesenchymal phenotype. This process is particularly relevant in epithelial cancers, in which normal epithelial cells, which are polarized along their apical–basal axis and are tightly connected to each other, undergone a phenotypic transformation characterized by loss of apical–basal polarity and acquisition of migratory characteristic that increase invasion and mobility. EMT program is regulated by EMT-transcription factors (i.e., Snail, Twist, and Zeb families) and comprises the coordinated upregulation and downregulation of so-called EMT genes among which there are several ECM and cell–cell adhesion proteins (124–128).

Periostin has been shown to promote EMT in several types of cancer cells. For example, Hu et al. showed that recombinant POSTN induced EMT in A549 and CL1-0 lung cancer cells. POSTN induced upregulation of mesenchymal genes such as N-cadherin, Vimentin, Twist, and Snail and downregulation of E-cadherin. The effects of POSTN were mediated by activation of ERK and p38 pathways and by downregulation of miR-381 expression, a miRNA targeting Twist and Snail mRNAs (39). POSTN has been also shown to induce EMT in prostate cancer cells. In this case, POSTN overexpression in PC3 and DU145 prostate cancer cells promoted cell proliferation, invasion, and migration. POSTN increased the expression of EMT-associated factors and activated Akt and GSK-3β pathways. Interestingly, TGF-β increased POSTN and Twist expression, suggesting that POSTN is a mediator of TGF-β-induced EMT (40). Curiously, POSTN has been also shown to inhibit EMT in others cancer cells. Thus, Kim et al. studied the effect of POSTN on EMT and cell invasiveness in bladder and prostate cancer cell lines and found opposite effects. Particularly, POSTN upregulated E-cadherin expression and suppressed cell invasiveness in bladder cancer cells while the opposite effects were observed in prostate cancer cells, suggesting that POSTN effects regarding EMT are tumor cell-dependent (113).

Several studies suggest that POSTN can act facilitating the interaction between cancer cells and the tumor niche to promote cell migration. These interactions are mainly mediated by interactions with receptors of the integrin family. For example, POSTN has been shown to support adhesion and migration of ovarian epithelial cancer cells by interacting with α_v_β_3_ and α_v_β_5_ integrins and thus cells cultivated on POSTN showed more motility than cells cultivated on fibronectin (13, 111). Orecchia et al. demonstrated that proliferation of a melanoma cell *in vivo* was inhibited by the addition of antibodies directed against an epitope of POSTN involved in the interaction with α_v_β_3_ and α_v_β_5_ integrins, indicating that this interaction is critical for tumor growth (111). In addition, ectopic expression of POSTN in the non-metastatic 293T cells increased cell migration, invasion, and adhesion and required signaling through α_v_β_5_ integrins and EGFR (129). Interestingly, inhibition of EGFR signaling was also shown to attenuate POSTN-mediated cell migration and invasion in esopahageal squamous cell carcinoma (114) supporting a relationship between POSTN and EGFR pathway.

Activating invasion is other critical step in the progression of malignant cancers. In this step, metalloproteinases play an important role by degrading different components of the extracellular matrix favoring the invasion of cancer cells. In this sense, POSTN has been shown to increase the activity of matrix metalloproteinase-2 and metalloproteinase-9 and promote migration and invasion in renal cell carcinoma cells (108, 109).

Finally, several studies indicate that POSTN can play a pivotal role in conditioning pre-metastatic niche and the formation of metastasis themselves. Fukuda et al. showed that the subcutaneous injection of osteoblasts that secrete large amounts of POSTN *in vivo* attracted remotely transplanted melanoma cells to the site of injection. By contrasts, osteoblasts in which POSTN expression was suppressed by shRNA showed a greatly reduced ability to promote metastasis (112). Malanchi et al. showed that infiltrating tumor cells are able to induce POSTN expression in the secondary target organ and that this ability is crucial for metastasis formation. Thus, the number of lung metastasis in an animal model was reduced in POSTN-null mice compared to wild-type control mice, suggesting that POSTN was a main mediator of metastasis (115). In addition, CAF-derived POSTN, induced by TGF-β3, was able to accelerate the migration and invasion of head and neck cancer cells (121). Taken together, these studies suggest that POSTN can act as a chemoattractant for cancer cells and that it is a key factor in metastatic colonization by conditioning of the premetastatic niche. Interestingly, POSTN could also be sent to the secondary metastatic sites by exosomes and thereby promote metastasis by conditioning the microenvironment of the target tissue before the arrival of tumor cells (120). In this sense, Vardaki et al. showed that the protein content of exosomes derived from metastatic human breast cancer cell lines was markedly different from that produced by non-metastatic cell lines. Specifically, exosomes secreted by metastatic cell lines showed a proteomic profile characterized by a high concentration of adhesion proteins such as β-Catenin, N-Cadherin, integrin-α2, integrin-β1, and POSTN. Remarkably, the characterization of exosomes isolated from the plasma of patients with metastatic or non-metastatic breast cancer showed similar characteristics to those observed in the metastatic and non-metastatic cell lines. These findings suggest that POSTN could serve as a biomarker for metastatic disease (120).

### Inducing Angiogenesis

Periostin has been shown to act as a potent pro-angiogenic factor. Breast cancer cell lines overexpressing POSTN promote tumor angiogenesis *in vivo* by upregulating the VEGF receptor Flk-1/KDR in endothelial cells through activation FAK signaling *via* integrin-αvβ3 (119). In addition, POSTN expression in head and neck cancer has been shown to correlate with VEGF-C expression, both in tumor tissue and serum. POSTN promoted tube formation of lymphatic endothelial cells, which was mediated by Src and Akt pathway (122).

### Resisting Cell Death

As previously mentioned above, POSTN acts as a prosurvival factor in several cellular contexts. POSTN has been shown to counteract hypoxia-induced apoptosis in cancer cells. For example, cancer cells that overexpressed POSTN were resistant to deferoxamine treatment, which mimic hypoxia (32). Aukkarasongsup et al. showed that periodontal cells in which POSTN expression was silenced using siRNAs were more susceptible to hipoxia-induced apopotosis than cells overexpressing perisotin. These effects were mediated by regulation of HIF-1α levels under hipoxic conditions (130). Furthermore, Liu et al. showed that POSTN expression was significantly increased in hepatocellular carcinoma cells under hypoxia. The regulation of POSTN was mediated by HIF-1α in such a way that HIF-1α depletion blocked the upregulation of POSTN induced by hipoxia. Interestingly, hypoxia-induced POSTN make the cells more resistant to arsenic trioxide treatment, and conversely, POSTN downregulation enhanced the anticancer effect of arsenic trioxide (123). In conclusion, POSTN could play an important role in the resistance to apoptosis under hypoxic conditions.

### Avoiding Immune Destruction

Modulation of immune system is a key element in cancer progression. In glioblastoma, glioma stem cells secret POSTN that contribute to recruit tumor-associated macrophages (M2). Thus, Zhou et al. showed that the disruption of POSTN in glioma stem cells *in vivo* was able to reduce the recruitment of tumor-associated macrophages and inhibit tumor growth. Therefore, the interplay between glioma stem cells and tumor-associated macrophages can be modulated by POSTN and may be critical to promote glioblastoma tumor growth (118).

## POSTN as a Therapeutic Target

Since POSTN plays multiple roles in cancer progression, targeting POSTN can be an attractive therapeutic approach. Several studies carried out in preclinical models suggest that blocking POSTN could be an attractive strategy to treat cancer. For example, Lee et al. used benzyl-d(U)TP-modified DNA aptamers (PNDAs) directed against human POSTN to treat breast cancer cell tumors in a xenograft mouse model. This strategy efficiently blocked tumor growth and cell dissemination to other organs (131). On the other hand, Kyutoku et al. used an anti-POSTN antibody (PN1-Ab), targeting the conserved exon 17 of POSTN, to treat mice of a lung metastasis model. Administration of this antibody significantly inhibited the growth of the primary tumors as well as the number of lung metastasis (132). Similar results were obtained by Zhu et al. using a monoclonal antibody (MZ-1) directed against POSTN in an *in vivo* model of ovarian cancer. In this case, *in vivo* administration of the antibody also produced a reduction in the number of metastasis (133).

## Concluding Remarks

The accumulated knowledge about the relationship between POSTN and cancer in the last two decades indicate that this matricellular protein play an important role in cancer development and progression, beyond its implications in the homeostasis of specialized tissues (7). The fact that POSTN can modulate the hallmarks of cancer suggests that this multifaceted protein can have implications for cancer diagnosis and prognosis and, more importantly, for the development of therapies targeting POSTN function. Future research should be directed to consolidate some findings and explore new ones, particularly in the field of prognostic markers and therapy.

While it seems clear that POSTN is overexpressed in many tumors and that this overexpression is frequently associated with poor prognosis, data are not probably enough conclusive to stratify different patients groups in terms of clinical response. In this sense, quantification of tumor POSTN levels in large cohort of patients treated homogenously in the context of well-controlled clinical trials will be necessary to confirm if tumor POSTN could be used as a reliable prognostic marker. Standardization of the immunohistochemistry techniques used to detect POSTN in the tumor tissues (for instance, antibodies and methods of quantification) will also be necessary to reach solid conclusions. Several laboratories have studied the levels of POSTN in serum and its correlation with tumor burden and prognosis. In this case, the results are more complex and there are not clear conclusions about the usefulness of POSTN levels in serum as a prognostic marker. Again, studies in larger cohorts could be necessary to clarify this question.

In addition to its ability to predict outcome, and perhaps for this, POSTN has been shown to induce resistance to some chemotherapeutic drugs in determined cancer cell types. If this is true in the clinical setting must still be determined, again in the context of observational clinical trials. This association between POSTN and resistance to anticancer drugs suggests that could be necessary design new combinational therapies in determined types of cancer to precisely circumvent the effect of the POSTN. Evidently, more basic and translational research, specifically in clinical relevant animal models (i.e., patient-derived xenografts) must be performed before translating it to the clinic. Furthermore, the relationship between POSTN and the proteins involved in the multidrug resistant phenotype in cancer (i.e., ABC family of transporters) should be also studied in more detail.

One of the most important finding is the implication of POSTN in the metastatic process, including different aspects such as the EMT, cell migration, and “education” of the metastatic niche. The idea that tumor cells or tumor stromal cells can remotely send a protein such as POSTN to prepare the metastatic niche is really exciting. Interestingly, the current knowledge strongly supports this possibility. Thus, several studies performed in animal models have demonstrated that POSTN can be a key determinant player in the “education” of the metastatic niche. Since the consequences of this finding could have a great clinical impact, more research is necessary about this topic. One of the unsolved questions is to know how POSTN reach the metastatic niche. One interesting possibility is that POSTN produced by tumor stromal cells or in some cases by the tumor cells themselves was sent to metastatic niche encapsulated in circulating exosomes. In this case, interference with the transport process could be of therapeutic interest. Comprehensive characterization of these exosomes at the molecular level will be necessary to determine if these exosomes have some characteristic potentially targetable. In addition, detection of POSTN levels into circulating exosomes could predict the risk of metastasis in clinically localized tumors, which could be exploited to direct patient stratification.

Finally, we know few about the relationship between POSTN and immune system. Since POSTN seems to play a role in maintaining the hematopoietic niche, it is probable that it could also play a role in modulating immune cell activities in tumors. For example, it is unknown if POSTN expression is associated to “cold” or “hot” tumors from an immunological point of view.

In summary, POSTN is a multifunctional protein deregulated in many solid tumors, which modulates tumor cell behavior in many ways. Next years will provide us with new findings about the interesting role of this protein in tumor development and progression and its relationship with new therapeutic approaches.

## Author Contributions

LG-G and JA wrote the manuscript and designed the figures and tables. JA corrected and supervised the manuscript.

## Conflict of Interest Statement

The authors declare that the research was conducted in the absence of any commercial or financial relationship that could be construed as a potential conflict of interest.
